# New York Letter

**Published:** 1881-03

**Authors:** 


### Domestic Correspondence.

---

### ARTICLE VIII.

### NEW YORK LETTER.

EDITORS JOURNAL AND EXAMINER:

Everyone who has the cause of medical education at heart will be disappointed and discouraged at the news that Bellevue Hospital Medical College has given up its three-year course. This it has done without giving the new system even a year's trial. The Faculty met in solemn convocation with the students a week or two ago. The Dean then arose and announced in almost plaintive tones that the College had had only fifty new students since last fall, when the new regulations went into force. The smallness of this number alarmed the Faculty. If it were not increased, the College would have to stop. To avoid even the possibility of such direful calamity, the old plan of a two-year course would be resumed. This back-down, after so very short a trial will certainly, and justly, be condemned by everyone. "If it were so soon to be done for, I wonder why it was begun for." There is no excuse for a return to the old system without having made a longer trial of the new. This action leaves the future of medical education in New York in a very unpromising condition. The two leading motives in such education now are these: The Colleges want to make all the money possible; the students want to get their degrees in as short a time and for as little money as possible. It should be said in justice to the College of Physicians and Surgeons, however, that their examinations are really quite rigid, and that a student can not get a degree without having stood a tolerably strict test of his proficiency.

One of the notable events of the present month was the annual meeting of the Academy of Medicine, when Dr. Fordyce Barker delivered his inaugural address. Other ceremonies filled up the programme, the most attractive one being a supper, at which the loving-cup was passed around, and a good many other cups followed in its wake. The attendance was large and the conviviality great. Dr. Barker's address was not a powerful effort, and hardly worthy of his powers. He spoke at his best when he condemned the narrow antagonism which regular physicians had so long shown towards homœopathy, and he thought that if this unreasonable exclusiveness and opposition on our part were withdrawn, homœopathy would soon sink to its proper level. He referred to the enviable social and financial position of (successful) New York physicians, and gave some instances of the liberality and appreciation of their patients. One physician in this city had received a New Year's present of $10,000. Several others had received somewhat smaller sums. Dr. Barker did not refer to the pecuniary status of those not in a fashionable practice. After his address, a number of prominent gentlemen were called on for supplementary remarks. It was disheartening to see the utter incompetence of distinguished Professors to make an impromptu speech. Still they all knew enough to say that they felt "gratified at the honor," but would prefer to remain quiet. As this was during the *mauvais del quart d'heure* before supper, their silence was after all felt to be the truest eloquence.

There seems to be a return in this city to the times of the Bacchantes and of those ingenuous days when processions in honor of the god Priapus marched through the public streets displaying the symbols of their worship. Any one walking along Broadway just now will encounter here and there a miserable wretch bearing aloft a flaming placard inscribed with this legend: "Use Damiana Bitters, Extract of Damiana, the Great Aphrodisiac Remedy and Stimulant to the Sexual Organs of Both Sexes." This advertisement is blazoned before the eyes of men down-town and of ladies up-town. It is little better than the advertisement of a brothel, and will doubtless increase business in that quarter. Not that damiana is an aphrodisiac, however. It neither excites nor increases erotic feelings except in rare cases. I have known as much as three ounces to be given daily without its affecting the sexual organs. It is, however, a tonic to the spinal cord, if we may use the term, and it is very useful in atony of the bladder.

While on the subject of foreign poisons I am inclined to furnish you with the results of an experiment with cannabis indica, made while house-physician at Bellevue Hospital.

### TOXIC EFFECTS OF CANNABIS INDICA.

The preparations of cannabis indica are so variable in strength, particularly in hospitals, that it used to be considered rather a duty to make occasional trials of their quality. Three patients, to whom drachm doses of the tincture were given, were made quite delirious by it, the delirium ending in some relief of pain and in a heavy sleep. Two of these patients were nervous women and the third was a man with locomotor ataxia. Therefore the results were not sufficiently definite and satisfactory. Determined to get more exact results, one evening at 7:30 p. m., I took a drachm dose of the tincture. At 9 p. m., feeling no especial effect, I took a drachm and a half more, making two and a half drachms in all. At about 10 p. m., while writing, I felt a mistiness over my eyes; I could not carry on any train of thought, and was obliged to stop my work. I was restless, and at length went up stairs to a friend's room, where several persons were sitting about. I began to feel some exhilaration. It was very easy to begin laughing and then very hard indeed to stop. There was an indescribable sensation of strangeness. I should have liked to have been introduced to myself and assured by the *alter ego* that I was all right, for I did not feel so. Soon I felt a numbness and compression on my head as though a long, slender-fingered hand were on it. This sensation passed to my body and extremities. It was a sort of tingling pressure, and not very pleasant. I could see and hear well. My throat became dry, and during the act of swallowing it seemed like an enormous cavern. I began to feel the drug more and more strongly. I was losing confidence in my power to control myself and keep my mind straight. I stopped talking, not knowing what conversational or emotional absurdity I might drop into. I felt as though I were isolated, set off from the others in the room, and that they were watching me intently and curiously, expecting some very interesting, or rather ridiculous, physiological effect to develop. This was, to some extent, actually the case. Finally I left the room, intending to go to bed. I did not feel a natural sleepiness, but rather a heaviness and inability to control my thoughts and take care of my body. My sensations were so strong that I concluded to walk out a little way and see if it would not clear things up a little. I walked without difficulty; was not dizzy, though my gait was at times uncertain. On coming back I felt a tingling and dragging weight in the calves of my legs. My mind was now in a very muddy and yet flighty state. My attention was constantly wandering off; I had no vivid fancies, but was wondering and speculating on this and that; I could only come back to my actual surroundings by an effort; my heart beat rapidly; my throat grew drier; my skin was wet with perspiration; I had no anæsthesia of the skin. A person came in to see me. I was rational, but seemed to be absent-minded and to think slowly. The feeling of heaviness and oppression grew on me. I was obliged to lie down, and at about 11:30 p. m. went to bed. Then came the visions; I felt as though borne up and up into space by some invisible, irresistible power. I went whirling round and round, up and up, in unending rotation, yet very gently and softly. At the same time crowds of faces and forms appeared before me; beautiful colors, shifting figures, innumerable shapes, gorgeous landscapes and tropical foliage came and went in endless panorama. By a great effort of will I could turn in bed and open my eyes; the visitors disappeared; then my eyes closed and they came again in continued succession and infinite variety. All the while I had the same feeling of being rolled on and on through an endless empyrean. These feelings were not pleasant, in fact there was an indefinable sense of oppression with them. I felt as though the drug had me in its power, and I wasn't at all sure that I had not taken too much of it. I had no sense of the prolongation of time, and none of the "double consciousness," which the drug is asserted to give. Fifteen or twenty minutes after going to bed I was roused to attend to a patient. I was able to get up, appreciate the fact that he had a urethral chill and give him M. x of Majendie and order some other needed measures, though it required a strong effort and close watching of myself. After returning to bed my visions and atmospheric flights ceased. Later, vomiting came on, and after that I fell into a heavy, dreamless sleep, from which I did not awake for eight hours. The next morning I felt a great deal of heaviness and *malaise*. My appetite was very good, however. I should say that as far as my experience goes, the delights of the hashish eater are illusive. The sensations are oppressive, the visions unsatisfactory, and if one must intoxicate himself he had better do it in the conventional way, with a reliable distillation of cereal products. As far as the practical usefulness of the drug is concerned as a hypnotic or anodyne, no one of the house staff was able to report any definite results, and my own subsequent experience was that it is an extremely unsatisfactory agent. It is quite slow in its action also.

The foregoing account was written shortly after the experiment, and it strikes me now that there is in its rhetoric a touch of the gorgeousness seen in the visions. But the sensations when one is thoroughly drugged with hashish are very powerful, and on coming out from them a person is apt to feel that language is quite inadequate for their expression.

Dr. George M. Beard has quite stirred the town with his experiments in mesmerism, or trance. He has a number of trance-patients whom he summons to his office two or three times a week and then brings out in them the various and curious phenomena characteristic of the condition. The interest in the subject is certainly spreading, and, as now studied, there may some results of practical value arise in mesmeric methods. They have indeed already been used practically in some of the dispensaries of the city, and have supplemented medicines in rheumatic, neuralgic, nervous and joint affections. It is said that there are more susceptible persons in the West than on the sea-board, and experiments on trance-patients will soon be undertaken there. The method, adopted by professionals, of testing the susceptibility of persons to trance, is to make the patient first gaze steadily at the mesmerizer while he places his hand on the forehead and makes gentle strokes with the thumb down towards the bridge of the nose. The patient is told to close his eyes and is gradually but firmly impressed with the belief that the manipulation will prevent the opening of the eyes. If susceptible it will be found in a few moments that the patient really cannot open his eyes, and then he is in a condition of trance and can be manipulated at the operator's will. The marked features of the condition are that the will of the mesmerized person is held in abeyance, and that he responds in every way to the suggestions of the operator. He sees, feels, hears and imagines whatever is suggested to him, and he does many things which show that his nervous power is greatly concentrated in the directions that are suggested to him. A most striking and demonstrative test of the reality of the phenomena is the increased muscular excitability that is developed very often. By pressing upon the "motor points" with a blunt point the contractions of the proper muscles ensue. Thus pressing on the motor point of the pronator *radii tires* will cause pronation; pressure on the motor point of the abductor *minimi digiti* will cause abduction of the little finger, and so on. Each particular muscle can thus be picked out just as is done with the battery to a patient in a normal condition.

Some very interesting and striking therapeutical results are now being claimed for static electricity. Dr. W. J. Morton is, in particular, trying to revive the use of this form of electricity, and he certainly appears to be justified in his enthusiasm over it. The apparatus used is like that of Charcot, and is very much larger than any heretofore employed by electro therapeutists. General tonic and sedative effects are gotten from it. But in addition, it seems to be peculiarly efficacious in old rheumatic or in neuralgic cases where there is probably some inflammatory effusion or adhesion. By the use of what is called the "electrical wind," conjunctivitis has been aborted also; so at least it is said. Excellent results follow in paralysis, and often much better results than the older forms of electricity give. All this, of course, is only one side of the matter. It may turn out, that on the whole not much more can be gotten from the sparks than from galvanic currents. I have, however, seen some of the results referred to, and cannot but think that static electricity ought to be tried again with better instruments than have so far been used.

The news in literary quarters is not very great. I hear, however, that Wm. Wood & Co. will soon publish a Supplement to

Ziemssen's Cyclopedia, which will bring everything in that treatise down to date. It will be published as a single volume, and will be entirely separate from the Cyclopedia. The same firm will soon publish a work on Histology and Microscopic Technology, which will undoubtedly attract much attention. It is edited by Dr. Thomas E. Satterthwaite, of this city, president of the Pathological Society and well known by his occasional contributions to pathology and histology. The book is written by different American histologists, and will represent concisely not only what Germany has done, but what American investigators have accomplished also.

NEW YORK CITY, Feb. 15, 1881.

